# The Great Medical Association of the Southwest to Meet Next Month in San Antonio

**Published:** 1909-10

**Authors:** G. H. Moody

**Affiliations:** San Antonio, Texas


### The Great Medical Association of the Southwest to Meet Next Month in San Antonio.

Letter from Dr. Moody, Chairman of Committee of Arrangements.

SAN ANTONIO, TEXAS, August 31, 1909.

*Dr. F. E. Daniel, Editor Texas Medical Journal, Austin, Texas.*

DEAR DOCTOR: As chairman of the general arrangements committee for the entertainment of the Medical Association of the Southwest, which will hold its annual meeting in San Antonio on November 9, 10 and 11, 1909, and as the Texas member of the publication committee of this association, I am requested by the chairman of this committee, Dr. L. Haynes Buxtom, of Oklahoma City, and by the secretary of the association, Dr. F. H. Clark, of El Reno, Oklahoma, to offer for publication in the medical journals of Texas a brief notice of this meeting, together with some information concerning the association, about which many of the Texas physicians may not be entirely familiar and especially those not living in North Texas, owing to the greater distance of the other parts of the State from those other States comprising this association.

The Medical Association of the Southwest covers the States of Missouri, Kansas, Oklahoma, Arkansas and Texas. Its membership numbers between six and seven hundred, the majority of whom are wideawake progressive physicians and surgeons, who are willing to put forth efforts for mutual scientific advancement.

This association was formerly the Tri-State Medical Association of Texas, Oklahoma and Indian Territories. At its annual meeting in Oklahoma City in 1906 there were present a body of representative physicians from the States of Missouri, Kansas and Arkansas, with a mutual request that we expand and include their States in one larger and greater association. Their request was accepted, the old Tri-State became the Medical Association of the Southwest, and its first annual meeting was held then.

In 1907 the annual meeting was held in Hot Springs, Arkansas, and in 1908 in Kansas City. At this meeting the association was invited to Texas and to San Antonio, and while many other important cities were clamoring for the next meeting, it was unanimously decided to go to San Antonio in 1909. These out of State visitors will be guests, not only of the physicians of San Antonio and Southwest Texas, but of every physician in Texas alike, all of whom are most cordially invited to come and be with us and help us entertain them, whether you are members of the association or not, and many from various parts of the State have already arranged to come. Efforts are being made for rates and for a special from the other States and an excursion from San Antonio to the City of Mexico is contemplated and rates already arranged. Any physician who is a member of the State Medical Association may become a member, either now or at the meeting, by application to the secretary, Dr. F. H. Clark, El Reno, Oklahoma.

As its name implies, this association is for the coming together of a body of physicians whose labors and interests are in a measure similar, and represents a great and rapidly developing territory, the great Southwest, whose future promises many things, medically as well as otherwise, among which is the greatest auxiliary medical association of the American Medical Association, with which it is in full harmony and accord.

Dr. William M. Welch, President of the American Medical Association, has promised to be here if possible; Dr. W. L. Rodman, of Philadelphia, will be here with an oration on "Surgery"; other prominent men from the East are expected, and prominent men from the other States which comprise the association will be here, and it will be profitable and pleasurable for any physician to attend.

Yours very truly,

G. H. Moody.

